# Genetics of Celiac Disease in Southern Brazil: High‐Resolution HLA Haplotypes and Non‐HLA Polymorphisms

**DOI:** 10.1111/tan.70972

**Published:** 2026-09-26

**Authors:** Fernanda Vitório da Silva, Valéria Bumiller‐Bini Hoch, Eduardo Delabio Auer, Priscila Ianzen dos Santos, Noah Cline, Luana Caroline Oliveira, Jennifer Elisabeth Hundt, Michael Wittig, Andre Franke, Paul J. Norman, Angelica Beate Winter Boldt

**Affiliations:** ^1^ Laboratory of Human Molecular Genetics (LGMH), Department of Genetics Federal University of Paraná (UFPR), Centro Politécnico, Jardim das Américas, Curitiba Paraná Brazil; ^2^ Postgraduation Program in Genetics Federal University of Paraná (UFPR), Centro Politécnico, Jardim das Américas, Curitiba Paraná Brazil; ^3^ Postgraduation Program in Internal Medicine Federal University of Paraná (UFPR), Centro de Ciências da Saúde, Curitiba Paraná Brazil; ^4^ Department of Biomedical Informatics University of Colorado Austin Colorado USA; ^5^ Luebeck Institute of Experimental Dermatology University of Luebeck Luebeck Germany; ^6^ Institute of Clinical Molecular Biology (IKMB), Christian‐Albrechts‐University of Kiel Kiel Germany

**Keywords:** Brazilian population, celiac disease, genetic susceptibility, HLA genotyping

## Abstract

Celiac disease (CeD) is an autoimmune enteropathy triggered by gluten ingestion in genetically predisposed individuals, who frequently present HLA‐DQ2 or HLA‐DQ8 haplotypes. To examine the genetic architecture of CeD in the admixed South Brazilian population, we sequenced the HLA alleles to high resolution and genotyped 16 non‐HLA polymorphisms, previously identified through genome‐wide association studies of Europeans in 171 CeD diagnosed patients and 195 controls. Controls had no CeD diagnosis, no first‐degree affected relatives, and were negative for anti‐TTg autoantibodies. As expected, we observed a predominance of *HLA*‐DQA1*05:01:01~DQB1*02:01:01 (DQ2.5) and *HLA*‐DQA1*02:01:01~DQB1*02:02:01 (DQ2.2) haplotypes among patients (51.7% and 40.2%, respectively). The ancestral 8.1 haplotype of HLA Class I and II alleles presented the highest odds of developing CeD (OR = 6.54, pcorr = 0.000065). By contrast, *HLA*‐DQA1*01:02:01, *HLA*‐DQB1*05:01:01, *HLA*‐DRB1*08:02:01 and *HLA*‐DRB1*13:01:01 were more frequent among controls (OR < 0.4, pcorr < 0.01). In contrast to results obtained in populations of predominantly European origin, HLA‐DQ8 frequencies did not differ between patients and controls. Among non‐HLA loci, rs6691768*G (g.61326191G > A) in *NFIA* was associated with protection (OR = 0.32, pcorr = 0.039) and rs653178*C (g.111569952C > T*)* (OR = 1.49, pcorr = 0.042) in ATXN2 was associated with susceptibility. This is the first high‐resolution HLA genotyping study of CeD in Brazil, providing a comprehensive overview of genetic susceptibility in an admixed population and highlighting new potential modulatory variants beyond the classical HLA loci.

## 1 Introduction

Celiac disease (CeD) is an autoimmune enteropathy induced by gluten ingestion in genetically susceptible individuals [1]. The seroprevalence of CeD in the general population ranges from 1.1% in Africa to 1.8% in Asia, with a global average of approximately 1.4% [2]. Nevertheless, higher frequencies have been reported in specific populations, such as 3.5% among Mennonites of Frisian/Flemish origin [3] and 5.6% in the Saharawi population [4]. CeD is more common in women, and its occurrence with other autoimmune diseases is not uncommon [5]. It is caused by a T cell‐mediated autoimmune reaction against tissue transglutaminase (an extracellular matrix enzyme), being triggered by gluten‐derived peptides. This reaction leads to mucosal damage and, eventually, intestinal villi atrophy [1, 5]. The diagnosis of CeD is classically based on a combination of the patient's clinical history, genetic tests, serological tests and duodenal biopsies [1].

Genetic predisposition is mainly conferred by HLA Class II haplotypes. The most strongly associated risk haplotype is a variant of HLA‐DQ2, constituted by HLA‐DQA1*05 and HLA‐DQB1*02 and called HLA‐DQ2.5, which is found in 90%–95% of individuals with CeD. The remaining patients with CeD carry HLA‐DQ8, the alleles HLA‐DQA1*03 and HLA‐DQB1*03:02 or another variant of HLA‐DQ2, called HLA‐DQ2.2 and formed by HLA‐DQA1*02:01 and HLA‐DQB1*02 [1]. HLA‐DQ2 and HLA‐DQ8 are common among the general population (around 30% of the population); however, only 3% of individuals with these HLA variants develop CeD [5]. Furthermore, there are differences in the risk of CeD between populations from different countries that cannot be explained by HLA genes [6]. In Paris and New York, the frequency of HLA‐DQ8 is higher in individuals in the control group than in celiac patients [7]. In Sweden, children are at an increased risk of developing CeD compared to children of populations with similar HLA genetic predisposition, indicating that risk may be influenced by variations in the environment and involvement of genes outside the HLA [6]. Genome‐wide association studies (GWAS) have identified 57 independent single‐nucleotide polymorphisms (SNPs) associated with risk outside the HLA in Europeans [8]. Additionally, five additional associations, amino acid position nine in HLA‐DPB1, HLA‐B*08:01, HLA‐B*39:06 and two SNPs, rs1611710 and rs2301226, explain 18% of the genetic risk related to HLA, independently of HLA‐DQA1 and HLA‐DQB1 alleles in individuals of European descent [9]. Environmental factors, in addition to genetic predisposition and exposure to gluten, such as viral infections and intestinal microbiota dysbiosis, may be involved in the development of CeD as well [4, 5].

The Brazilian population exhibits a high degree of admixture, reflected in the wide variation in the proportions of Native American, African and European ancestry among individuals, shaping a complex mosaic of genetic diversity [10]. However, data on the genetic profile of Brazilian celiac patients, particularly beyond the HLA‐DQ2.5 and HLA‐DQ8 haplotypes, remain limited. In this context, the present study aims to characterise the genetic profile of individuals from Southern Brazil diagnosed with CeD, with an emphasis on high‐resolution analysis of HLA variants and non‐HLA SNPs. The findings aim to contribute to a better understanding of the genetic basis of the disease across different population contexts, considering the high genetic diversity of the Brazilian population [10].

## 2 Materials and Methods

### 2.1 Population Sample

The study was approved by the Ethics Committee of the Health Sciences Sector of the Federal University of Paraná—CEP SCS‐UFPR, according to the Helsinki declaration, within the scope of the MedEpiGen Project: Preventive Medicine for Metabolic Syndrome (CAAE 55528222.9.0000.0102). Contact with participants and sample collection were conducted at the Celiac Association of Paraná (ACELPAR) in Curitiba, Brazil, between 2022 and 2024. Participation was voluntary and required the signing of an Informed Consent Form. Blood samples were collected from 366 admixed individuals of major European descent, including 171 patients clinically diagnosed with CeD through positive duodenal biopsy and 195 controls, serologically negative for anti‐tissue transglutaminase (anti‐tTG) IgA; both are not related (Table 1). The case group was formed by individuals with a previous medical diagnosis of CeD. The control group included individuals who declared themselves without CeD (subsequently confirmed by negative serological screening for CeD), without other autoimmune diseases, even in their family history (first degree relatives).

**TABLE 1:** Sample characterisation.

	CeD *N* = 171	CTR *N* = 195	OR	95% CI	*p*	pcorr
Age (years–min‐max)	40.6 (12–76)	41.9 (13–76)	0.99	0.98–1.01	0.38	
**Women (%)**	**90.6**	**78.5**	**2.66**	**1.46–5.06**	**0.002**	**0.011**
Major European ancestry^a^	70.2	73.1			0.2	
Mixed ancestry^a^	29.7	24.7				
Asian ancestry^a^	0	2.1				

*Note:* In bold: significant associations (*p* < 0.05). Based on the same data, an average European component of up to 34% and an average Native American component of up to 6% is expected for the individuals with mixed ancestry [11, 12]. Asian ancestry was self‐reported by Japanese immigrants. Age and sex proportions were analysed with univariate logistic regression, whereas self‐reported ancestries were compared using Fisher exact test.

Abbreviations: CeD, celiac disease; CI, confidence interval; CTR, control; OR, odds ratio; *p*, *p*‐value; pcorr, significant *p*‐values werer corrected *p*‐values using “False discovery rate” method.

^a^ Self‐reported. Based on HLA allelic frequencies previously determined for the Curitiba population (from which we also obtained the samples), European ancestry presents cc. 9% African and 5% Native American genetic component.

### 2.2 Serological Screening for CeD

We performed enzyme‐linked immunosorbent assay (ELISA) for detection of anti‐tTG IgA (EA1910‐9601A, Euroimmun, Germany) in serum samples. According to the manufacturer's instructions, samples were considered positive with antibody concentrations equal to or higher than 20 RU (relative units)/ml, using calibration curves, positive and negative controls. The reading was carried out on a spectrophotometer using a filter with a wavelength of 450 nm and a reference filter of 620 nm.

### 2.3 HLA Library Preparation and Sequencing

We isolated genomic DNA from whole blood using Wizard Genomic DNA Purification kit protocol (Promega, Madison, WI, USA). The DNA was resuspended in ultrapure water and stored in a freezer at −20°C. The quantity and quality of DNA were assessed using a NanoDrop spectrophotometer.

DNA was prepared for high throughput targeted sequencing of the *HLA* genes as described by Norman et al. [13]. Briefly, DNA was enzymatically fragmented and end‐repaired using the NEB Next Ultra II Module (New England Biolabs, Ipswich, MA). A dual side size selection was performed to select ~900 bp fragments using AmpureXP beads (Beckman Coulter, Indianapolis IN). Post size selection, unique molecular indices (UMIs) (Integrated DNA technologies, Coralville, IA) were appended to each sample via PCR using the KAPA HiFi HotStart ReadyMix (Roche Diagnostics Corporation, Indianapolis, IN), and PCR primers were removed using AmpureXP beads (Beckman Coulter, Indianapolis, IN). The amplified, indexed, DNA libraries were quantified and pooled into batches of 96 at equimolar quantities for enrichment.

To generate sufficient sequence data for the highly polymorphic HLA genomic regions, we utilised our previously described enrichment method [13], with modifications as described by Farias et al. [14]. Briefly, biotinylated probes specific for the HLA Class I or HLA Class II genes were hybridised to the prepared libraries. The hybridised DNA fragments were isolated using streptavidin beads and amplified based on the Illumina Nextera Capture protocol (Illumina, San Diego CA). Captured libraries were sequenced using an Illumina HiSeq with 2 × 250 bp read length. The enriched libraries were pooled at equimolar quantities prior to sequencing.

HLA alleles were determined from the sequence data using a combination of GenDx NGSEngine version 3.1.0.32783 and Omixon HLA Explore, using the IPD IMGT/HLA 3.53.0 database as reference [15]. To improve alignment quality and remove low‐quality sequence data, Fastq files were prefiltered using the Java‐based Omixon HLA Twin Development Fastq filter tool.

### 2.4 Typing of Non‐HLA Variants

In a search in the GWAS Catalog [16], 16 non‐HLA SNPs previously identified as being associated with CeD (Table 2, Table S1) were selected for genotyping. All selected SNPs have a minor allele frequency (MAF) higher than 10% in European populations, according to Ensembl Genome data [19]. Genotyping of selected variants was performed using the iPLEX MassARRAY platform (Sequenom, San Diego, CA). Genotyping quality was analysed using the MassARRAY Typer 4.0 software (Sequenom).

**TABLE 2:** CeD predisposition SNPs selected for IPLEX.

Genes	SNP	HGVS^a^	Location	MAF (%)
NFIA	rs6691768‐G [17]	NC_000001.11:g.61326191G > A	Intron variant	G: 41
TNFSF18	rs12142280‐A [8]	NC_000001.11:g.172895512 T > A	Intron variant	A: 18
RGS1	rs2816316‐C [17]	NC_000001.11:g.192567683C > A	Intron variant	C: 18
RGS1	rs1359062‐C [8, 18]	NC_000001.11:g.192572342C > G	Intron variant	C: 19
IL18RAP, SLC9A4	rs990171‐A [8]	NC_000002.12:g.102470310A > C	Intergenic variant	A: 22
PUS10	rs13003464‐G [8, 17]	NC_000002.12:g.60959694A > G	Intron variant	G: 36
IL12A	rs1353248‐T [8]	NC_000003.12:g.159905770C > T	Intergenic variant	T: 28
BLTP1	rs13151961‐G [14, 17]	NC_000004.12:g.122194347A > G	Intron variant	G: 16
LINC03004	rs17264332‐G [8]	NC_000006.12:g.137684378A > G	Intron variant	G: 17
TAGAP	rs1738074‐T [17]	NC_000006.12:g.159044945 T > C	5 prime UTR variant	T: 43
TAGAP	rs182429‐A [8]	NC_000006.12:g.159048542A > G	Intron variant	A: 44
ZMIZ1	rs1250552‐G [8, 17]	NC_000010.11:g.79298270A > G	Intron variant	G: 44
MIR4491, POU2AF3	rs7104791‐T [8]	NC_000011.10:g.111326133 T > C	Intergenic variant	T: 21
ATXN2	rs653178‐C [17]	NC_000012.12:g.111569952C > T	Intron variant	C: 47
UBASH3A	rs1893592‐C [8]	NC_000021.9:g.42434957A > C	Splice donor region variant	C: 28
YDJC, UBE2L3	rs4821124‐C [8]	NC_000022.11:g.21625000 T > C	Regulatory region variant	C: 18

*Note:* NFIA—nuclear Factor I A; TNFSF18—TNF superfamily member 18; RGS1—regulator of G protein signalling 1; PUS10—pseudouridine synthase 10; SLC9A4—solute carrier family nine member A4, IL18RAP—interleukin 18 receptor accessory protein; IL12A—IL12A antisense RNA 1; BLTP1—bridge‐like lipid transfer protein family member 1; LINC03004—long intergenic non‐protein coding RNA 3004; TAGAP—T cell activation RhoGTPase activating protein; ZMIZ1—zinc finger MIZ‐type containing 1; MIR4491—microRNA 4491, POU2AF3—POU Class 2 homeobox associating Factor 3; ATXN2—ataxin 2; UBASH3A—ubiquitin associated and SH3 domain containing A; UBE2L3—ubiquitin conjugating enzyme E2 L3, YDJC—YdjC chitooligosaccharide deacetylase homologue.

Abbreviation: MAF, Menor Allele frequency in European populations by ensemble.

^a^ HGVS Nomenclature by Genome Reference Consortium Human Build 38 patch release 14 (GRCh38.p14).

### 2.5 Data Analysis

We used R package BIGDAWG V3.0.3 for HLA association analysis [20] and PLINK v1.1.9 [21] for genetic association testing in SNPs, using dominant, additive and recessive models. Hardy–Weinberg (HW) equilibrium was assessed in the control group using the exact test with a mid‐*p* value approach implemented in PLINK v.1.9. The genotype distribution of just one genetic variant deviated significantly from the expected values (*p* < 0.05) and was excluded from further analyses. The covariates were identified by univariate logistic regression test with R language version 4.3.2 [22]. Sex distribution differed significantly between groups (Table 1). Therefore, sex was included as a covariate in subsequent multivariate logistic regression models including HLA and SNP association analyses. Non‐HLA SNP associations found in the univariate analysis were further adjusted for the HLA haplotypes significantly associated with the disease, to detect possible HLA‐independent effects. *p* ≤ 0.05 was considered significant, after correction for multiple testing for False discovery rate [23]. The odds ratio was calculated with a 95% confidence interval. Statistical power was 70%–90% for the detection of small to medium effects (effect sizes of 14%–39%, respectively).

## 3 Results

### 3.1 HLA Alleles

We identified 11 HLA alleles associated with CeD predisposition, both in the dominant and additive models and after correction for multiple testing (pcorr < 0.05). As expected, the main genetic risk factors were the alleles that form the HLA‐DQ2.5 heterodimer: HLA‐DQA1*05:01:01 (OR = 4.94, 95% CI = 3.08–8.06, pcor *r* = 3.8e^−10^) and HLA‐DQB1*02:01:01 (OR = 5.03, 95% CI = 3.13–8.23, pcorr = 3.8e^−10^), both linked to HLA‐DRB1*03:01:01 (OR = 5.23, 95% CI = 3.24–8.61, pcorr = 3.8e^−10^). They were followed by the alleles that form the HLA‐DQ2.2 heterodimer: (HLA‐DQA1*02:01:01; OR = 2.88, 95% CI = 1.80–4.68, pcorr = 3.7e^−05^ and HLA‐DQB1*02:02:01; OR = 3.42, 95% CI = 2.14–5.56, pcorr = 1.5e^−06^), linked to DRB1*07:01:01 (OR = 2.72, 95% CI = 1.70–4.40, pcorr = 8.6e^−05^). Other alleles associated with CeD predisposition included HLA‐A*01:01:01, HLA‐B*08:01:01, HLA‐C*07:01:01, HLA‐DPB1*01:01:01 and HLA‐DPB1*17:01:01 (Table 3, Table S2).

**TABLE 3:** HLA alleles associated with predisposition and protection to CeD.

HLA allele	Freq %		Dominant model				Additive model			
	CTR	CeD	OR	95% CI	*p*	pcorr	OR	95% CI	*p*	pcorr
DRB1*03:01:01	17.9	51.1	5.23	3.24–8.61	2.9e‐11	**3.8e‐10**	3.79	2.47–5.96	2.7e‐09	**2.7e‐08**
DQB1*02:01:01	18.9	51.7	5.03	3.13–8.23	5.1e‐11	**3.8e‐10**	3.69	2.42–5.79	4.4e‐09	**2.7e‐08**
DQA1*05:01:01	18.9	51.7	4.94	3.08–8.06	6.6e‐11	**3.8e‐10**	3.64	2.39–5.68	4.7e‐09	**2.7e‐08**
DPB1*17:01:01	2.6	10.9	4.27	1.66–13.2	0.005	**0.006**	4.27	1.66–13.2	0.005	**0.006**
B*08:01:01	11.7	34.5	4.08	2.40–7.16	4.3e‐07	**1.5e‐06**	3.44	2.11–5.80	1.6e‐06	**5.4e‐06**
DQB1*02:02:01	17.9	43.7	3.42	2.14–5.56	4.2e‐07	**1.5e‐06**	3.06	1.96–4.86	1.4e‐06	**5.4e‐06**
DQA1*02:01:01	18.4	40.2	2.88	1.80–4.68	1.3e‐05	**3.7e‐05**	2.49	1.60–3.92	6.1e‐05	**1.7e‐04**
DRB1*07:01:01	18.9	39.7	2.72	1.70–4.40	3.5e‐05	**8.6e‐05**	2.35	1.51–3.70	1.7e‐04	**4.1e‐04**
A*01:01:01	15.8	32.8	2.53	1.54–4.22	2.9e‐04	**5.5e‐04**	2.10	1.35–3.32	0.001	**0.002**
C*07:01:01	20.4	38.5	2.52	1.58–4.06	1.2e‐04	**2.6e‐04**	2.23	1.47–3.44	2.2e‐04	**4.7e‐04**
DPB1*01:01:01	16.3	29.3	2.06	1.25–3.44	0.005	**0.006**	1.96	1.26–3.12	0.003	**0.005**
DQA1*04:01:01	9.7	0.6	0.05	0.00–0.22	0.003	**0.004**	0.05	0.00–0.25	0.004	**0.005**
DRB1*08:02:01	6.6	0.6	0.07	0.00–0.38	0.012	**0.012**	0.07	0.00–0.38	0.012	**0.012**
DQB1*04:02:01	10.2	1.1	0.09	0.01–0.30	0.001	**0.002**	0.10	0.02–0.33	0.002	**0.003**
DQA1*01:02:01	33.2	17.2	0.44	0.26–0.71	0.001	**0.002**	0.43	0.26–0.68	5.0e‐04	**9.5e‐04**
DQB1*05:01:01	20.4	9.8	0.44	0.23–0.80	0.009	**0.010**	0.44	0.23–0.78	0.006	**0.007**
DRB1*13:01:01	15.8	7.5	0.40	0.20–0.78	0.010	**0.010**	0.40	0.20–0.74	0.006	**0.007**

*Note:* Logistic regression association tests were done with allele frequencies (additive model), frequency of homozygotes for the minor allele (recessive model) and summed frequencies of heterozygotes and homozygotes for the minor allele (dominant model). In bold: significant associations (*p* < 0.05); *p*‐value reported in scientific notation when *p* < 0.001.

Abbreviations: CeD, celiac disease; CI, confidence interval; CTR, control; OR, odds ratio; *p*, *p*‐value; pcorr, corrected *p*‐values using “False discovery rate” method.

Conversely, six alleles were associated with protection against CeD: HLA‐DQA1*01:02:01, HLA‐DQA1*04:01:01, HLA‐DQB1*04:02:01, HLA‐DQB1*05:01:01, HLA‐DRB1*08:02:01 and HLA‐DRB1*13:01:01. The strongest protective effect was observed for HLA‐DQA1*04:01:01 (OR = 0.05, 95% CI = 0.00–0.22, pcorr = 0.004) (Table 3).

Haplotype analysis confirmed and reinforced these associations. Among the risk haplotypes, the most notable were HLA‐DQ2.5: HLA‐DQA1*05:01:01~DQB1*02:01:01 (OR = 3.38, 95% CI = 2.24–5.17, pcorr = 9.5e^−09^), present in 51.7% of patients versus 18.4% of controls and HLA‐DQ2.2: HLA‐DQA1*02:01:01~DQB1*02:02:01 (OR = 2.71, 95% CI = 1.72–4.35, pcorr = 4.2e^−05^), observed in 40.2% of patients and 16.3% of controls. The complete haplotype HLA‐A*01:01:01~B*08:01:01~C*07:01:01~DPB1*01:01:01~DQA1*05:01:01~DQB1*02:01:01~DRB1*03:01:01 presented the highest risk (OR = 6.54; 95% CI = 2.44–21.99, pcorr = 6.5e^−05^), being detected in 14.4% of patients and only 2% of controls. In contrast, the haplotype HLA‐DQA1*04:01:01~DQB1*04:02:01 was associated with protection (OR = 0.05; 95% CI = 0–0.32; pcorr = 1.7e^−04^), a finding also confirmed in the presence of HLA‐DRB1*08 (Table 4).

**TABLE 4:** HLA haplotypes associated with CeD.

HLA haplotypes	OR	CTR (%) *N* = 195	CeD (%) *N* = 171	95% CI	*p*	pcorr
DQA1~DQB1						
*05:01:01~*02:01:01	3.38	36 (18.4)	90 (51.7)	2.24–5.17	5.3e‐10	9.5e‐09
*02:01:01~*02:02:01	2.71	32 (16.3)	70 (40.2)	1.72–4.35	4.6e‐06	4.2e‐05
*04:01:01~*04:02:01	0.05	18 (9.2)	1 (0.6)	0–0.32	2.9e‐05	1.7e‐04
DQA1~DQB1~DRB1						
*05:01:01~*02:01:01~*03:01:01	3.62	33 (16.8)	89 (51.1)	2.37–5.61	1.5e‐10	2.6e‐09
*02:01:01~*02:02:01~*07:01:01	2.62	32 (16.3)	68 (39.1)	1.66–4.2	1.1e‐05	6.6e‐05
*04:01:01~*04:02:01~*08:02:01	0.09	12 (6.1)	1 (0.6)	0–0.63	0.0039	0.018
A~B~C~DQA1~DQB1~DRB1						
*01:01:01~*08:01:01~*07:01:01~ *05:01:01~*02:01:01~*03:01:01	3.33	13 (6.6)	37 (21.3)	1.72–6.77	8.4e‐05	2.1e‐04
A~B~C~DPB1~DQA1~DQB1~DRB1						
*01:01:01~*08:01:01~*07:01:01~ *01:01:01~*05:01:01~*02:01:01~*03:01:01	6.54	4 (2.0)	25 (14.4)	2.44–21.99	1.3e‐05	6.5e‐05

Abbreviations: CeD, celiac disease; CI, confidence interval; CTR, control; OR, odds ratio; *p*, *p*‐value; pcorr, corrected *p*‐values using “False discovery rate” method; *p*‐value reported in scientific notation when *p* < 0.001.

**FIGURE 1:**
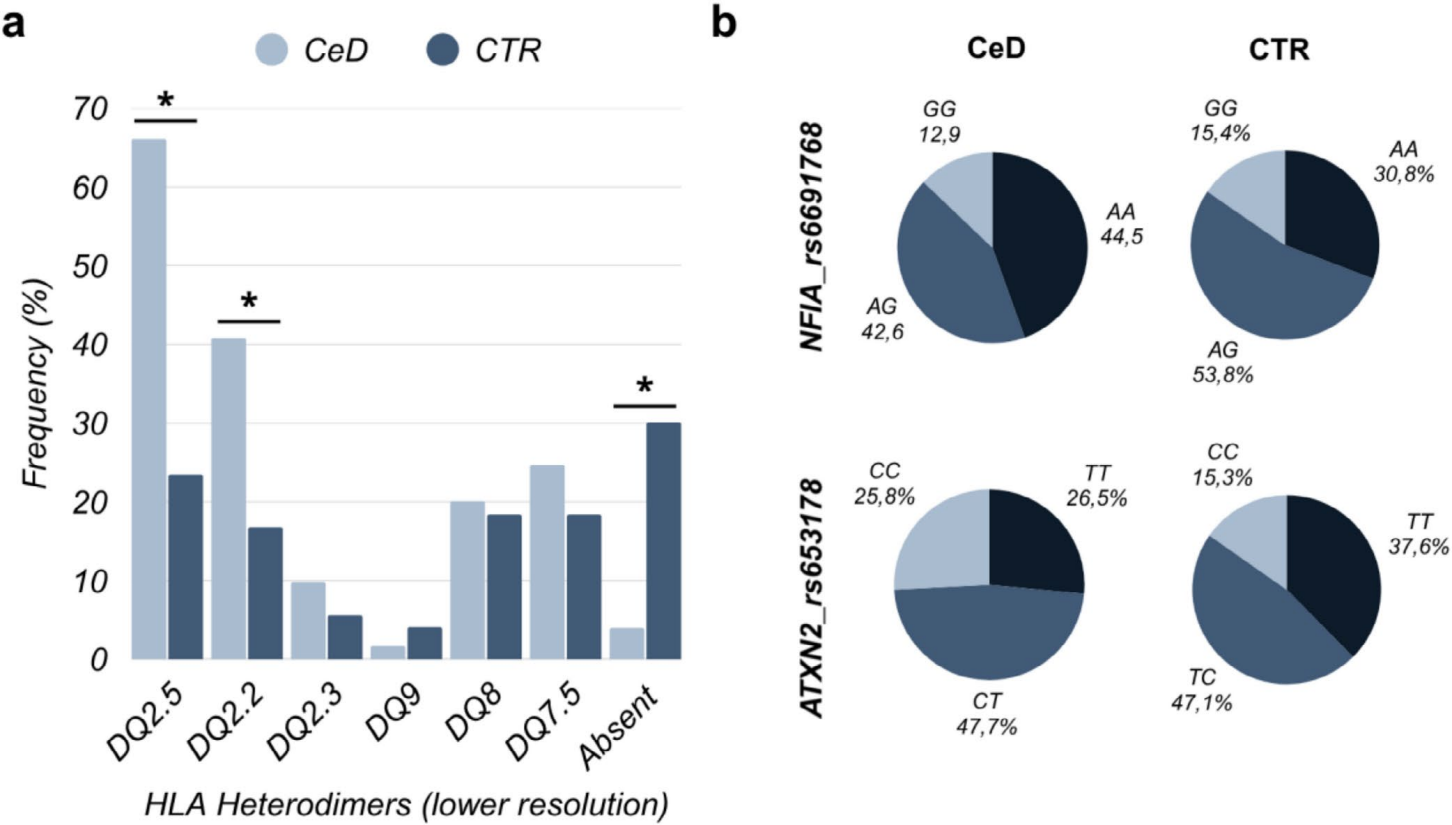
Genetic association with predisposition and protection to CeD. CeD, Celiac disease; CTR, Control; Absent, Individuals without the predisposing HLA‐DQ haplotypes; *p < 0.001; (a) bar chart containing HLA heterodimers analysed at lower resolution to compose the haplotypes cited in the literature as associated with CeD; (b) genotypic frequency of SNPs rs6691768 and rs653178 associated with CeD in patients and controls; NFIA—nuclear Factor I A; ATXN2—ataxin 2.

To compare with previously published results, we analysed our data at a lower‐resolution (one fiend), evaluating other heterodimers: HLA‐DQ2.3 (HLA‐DQA1*03~DQB1*02) [24], HLA‐DQ7.5 (HLA‐DQA1*05:05~DQB1*03:01) [25] and HLA‐DQ9 (HLA‐DQA1*03~DQB1*03:03) [26], reported in the literature (Figure 1a, Tables S3 and S4). The frequency of individuals presenting HLA‐DQ7.5 without HLA‐DQ2.2 or HLA‐DQ2.5 was higher among controls (OR = 0.21, 95% CI = 0.08–0.46, pcorr = 0.001), whereas frequencies of all others did not differ between controls and patients. On the other hand, HLA‐DQ7.5 accompanied by HLA‐DQ2.2 and HLA‐DQ2.5, increases the chance of developing CeD by 3.9 times (OR = 3.91, 95% CI = 1.70–10.1, pcorr = 0.007).

### 3.2 Non‐HLA SNPs Associated With Celiac Disease

Of the 16 non‐HLA SNPs analysed, one (rs1359062, g.192572342C > G) deviated from HW equilibrium and was excluded from the analyses. Analysis of variants revealed a significant association of two SNP with CeD, as well as trend associations for three SNPs (Table 5, Table S5), independently of the presence of predisposing HLA‐DQA1*04:01:01~DQB1*04:02:01, HLA‐DQA1*05:01:01~DQB1*02:01:01 and HLA‐DQA1*02:01:01~DQB1*02:02:01 HLA haplotypes. The variant rs6691768*G (g.61326191G > A), located in the Nuclear factor I A (NFIA) gene, was associated with protection against CeD (Figure 1b) in the dominant model (OR = 0.53, 95% CI = 0.31–0.92, pcorr = 0.039) and the variant rs653178*C (g.111569952C > T) in Ataxin 2 (ATXN2) was associated with susceptibility in the allele frequency model (OR = 1.49, 95% CI = 1.03–2.16, pcorr = 0.042).

**TABLE 5:** Associated SNPs with CeD.

GENE	SNP	MODEL	(%)		OR	95% CI	*p*	*pcorr*
			CTR	CeD				
*NFIA*	*rs6691768*	add	42.3	34.2	0.68	0.46–1.00	0.053	0.066
1p31.3	g < A	rec	15.4	12.9	0.80	0.37–1.69	0.6	0.6
		**dom**	**69.2**	**55.5**	**0.53**	**0.31–0.92**	**0.024**	**0.039**
ATXN2	*rs653178*	**add**	**38.9**	**49.7**	**1.49**	**1.03–2.16**	**0.033**	**0.042**
12q24.12	c < T	rec	15.3	25.8	1.69	0.88–3.31	0.12	0.14
		dom	62.4	73.5	1.71	0.99–2.99	0.057	0.072
*PUS10, REL*	*rs13003464*	add	43.0	49.4	1.40	0.98–2.03	0.069	0.086
*2p16.1*	g < A	rec	17.7	26.5	1.89	1.01–3.58	**0.049**	0.061
		dom	68.4	72.3	1.36	0.77–2.40	0.3	0.4
*ZMIZ1*	*rs1250552*	add	51.6	44.8	0.68	0.47–0.99	**0.047**	0.059
10q22.3	g < A	rec	24.8	20.6	0.67	0.36–1.25	0.2	0.3
		dom	78.3	69.0	0.55	0.30–0.99	0.050	0.062
*UBE2L3, YDJC*	*rs4821124*	add	19.7	22.9	1.42	0.91–2.24	0.12	0.2
22q11.21	c < T	rec	5.7	3.9	0.82	0.23–2.76	0.7	0.7
		dom	33.8	41.9	1.73	1.01–2.97	**0.047**	0.058

*Note:* Logistic regression association tests were done with allele frequencies (‘add’), frequency of homozygotes for the minor allele (‘rec’‐ recessive model) and summed frequencies of heterozygotes and homozygotes for the minor allele (‘dom’—dominant model). Sample size slightly differed for controls because of genotyping quality (*N* = 156–158 individuals), whereas for patients it remained the same (*N* = 158). The minor allele in our sample is given in lowercase; being the reference for the associations. In bold, significant associations (pcorr < 0.05). NFIA—nuclear Factor I A; ATXN2—ataxin 2; PUS10—pseudouridine synthase 10; ZMIZ1—zinc finger MIZ‐type containing 1; UBE2L3—ubiquitin conjugating enzyme E2 L3.

Abbreviations: CeD, celiac disease; CI, confidence interval; CTR, controls; MAF, minor allele frequency; Model, association tests; OR, odds ratio; pcorr, corrected *p*‐values using ‘False discovery rate’ method; SNP, single nucleotide polymorphism.

Additional SNPs, including rs13003464*G (g.60959694A > G) in the *Pseudouridine synthase* 10 gene (PUS10), rs1250552*G (g.79298270A > G) in *Zinc* finger MIZ‐type containing 1 (ZMIZ1) and rs4821124*C (g.21625000 T > C) in Ubiquitin conjugating enzyme E2 L3 (UBE2L3) exhibited a trend towards association that did not reach statistical significance after correction for multiple testing (pcorr = 0.05).

## 4 Discussion

Brazil is home to the largest population in Latin America and represents one of the world's largest recently admixed populations. Genetic ancestry varies across regions, with higher Native American ancestry in Northern Brazil, predominant African ancestry in the Northeast (~50%), and predominant European ancestry in the Southeast and South (> 70%) [10]. However, analyses based on HLA allelic frequencies demonstrated substantial differences between self‐reported ethnicity and genetic ancestry. An average African component of 9% and an average Native American (mostly Guarani/Kaingang) component of 5% were estimated for individuals of European descent in Curitiba, based on HLA allelic frequencies previously determined by our group [11, 12].

Given that HLA allele frequencies are strongly influenced by population ancestry, the predominantly European genetic background observed in Southern Brazil may contribute to the high frequency of CeD‐associated HLA haplotypes identified in our cohort. Our results confirm the central role of the HLA region in CeD genetic susceptibility, with emphasis on the HLA‐DQA1*05:01:01 and HLA‐DQB1*02:01:01 alleles composing the *HLA‐DQ2.5* heterodimer and HLA‐DQA1*02:01:01 and HLA‐DQB1*02:02:01 alleles composing the HLA‐DQ2.2 heterodimer, whose robust associations have already been consistently described in other populations [1]. We also found that the presence of the DQ2.5 heterodimer in trans with 2.2 increases the chance of developing CeD by 13 times (OR = 13.4, pcorr < 0.001). The high frequency of these alleles among patients reinforces their contribution to the antigenic presentation of gluten‐derived peptides and activation of the adaptive immune response. The finding of the extended haplotype 8.1 HLA‐A*01:01:01~B*08:01:01~C*07:01:01~DQA1*05:01:01~DQB1*02:01:01~DRB1*03:01:01, in addition to HLA‐DPB1*01:01:01, presented the highest risk (OR = 6.54) and corroborates previous studies [27] that describe the ancestral haplotype 8.1 as a classic marker of predisposition to autoimmune diseases, including CeD, in Europeans.

On the other hand, we identified HLA‐DQA1*01:02:01, HLA‐DQA1*04:01:01, HLA‐DQB1*04:02:01, HLA‐DQB1*05:01:01, HLA‐DRB1*08:02:01 and HLA‐DRB1*13:01:01 alleles associated with reduced CeD risk, as well as the related haplotypes HLA‐DQA1*04:01:01~DQB1*04:02:01. In Europeans, the HLA‐DQA1*01:02:01 allele has been previously described as associated with protection against Type 1 diabetes [28], while HLA‐DQB1*05:01 has been associated with protection against systemic sclerosis [29]. Nevertheless, HLA‐DRB1*08:02 has been associated with susceptibility to systemic lupus erythematosus in the Japanese population [30]. These results reinforce previous observations that certain alleles may reduce binding affinity to gluten peptides, conferring lower risk against the CeD‐autoimmune response, at least in European populations [31].

To enable comparison with studies previously conducted in Brazil, the data were also analysed at a lower resolution. In the Central‐West region, a frequency of 73% was observed for HLA‐DQ2.5, 12% for HLA‐DQ8, and 7% for the HLA‐DQ2.5*~*DQ8 combination in patients [32]. Similar frequencies are described in the Northeast region of the country (HLA‐DQ 2.5%–68.6%, HLA‐DQ8%–17.8%, and HLA‐DQ2.5~DQ8%–6.8%) [33]. These results are consistent with those obtained in our study for South Brazil, where the frequencies in CeD patients were 63.2% for HLA‐DQ2.5, 17.2% for HLA‐DQ8 and 2.9% for HLA‐DQ2.5~DQ8.

An additional relevant finding was the absence of a significant difference in the frequency of HLA‐DQ8 between controls (18.4%) and CeD patients (20.1%). This suggests that in the Brazilian population, the relative contribution of HLA‐DQ8 to susceptibility may be less pronounced. This is also observed in New York (33% in patients and 55% in controls) and in Paris (5.2% in patients and 6.2% in controls) [7]. The same was observed for HLA‐DQ7.5, HLA‐DQ9 and HLA‐DQ2.3, which showed no significant differences between cases and controls.

In addition to the HLA region, we detected two associations involving non‐HLA variants. The SNP rs6691768*G (g.61326191G > A) in *NFIA* was associated with a protective effect and the SNP rs653178*C (g.111569952C > T) in ATXN2 was associated with susceptibility effect. Polymorphisms located in the NFIA gene were found associated with several processes in at least 438 GWAS [19] associations, highlighting its pleiotropy. NFIA acts as a regulator of brown adipocyte differentiation [34], and there is evidence that it promotes the expression of genes related to mitochondrial oxidative phosphorylation while negatively regulating genes involved in inflammatory processes [35]. Nevertheless, the association with CeD is the only one with an autoimmune disease, reported in three different studies [17, 19]. On the other hand, the SNP rs653178 has been associated with several conditions, including autoimmune diseases and metabolic disorders [19].

Our study is the first to replicate former GWAS‐association results in Brazil. Only two of the 16 genotyped non‐HLA variants showed significant associations in our cohort. The reason for this difference is not clear but it may reflect the ethnic admixture of Brazilian populations. Many non‐HLA variants associated with CeD confer modest effect sizes compared with HLA risk alleles, limiting statistical power in smaller samples and rendering replication particularly sensitive to demographic heterogeneity. Additional variants, including rs13003464*G (g.60959694A > G) in the PUS10, rs1250552*G (g.79298270A > G) in ZMIZ1 and rs4821124*C (g.21625000 T > C) in UBE2L3, did not reach statistical significance after correction for multiple testing but showed suggestive evidence of association (pcorr = 0.055), which may warrant further investigation in larger cohorts.

This is the first study conducted in Brazil to investigate the association of HLA with CeD using high‐resolution sequencing. Previous studies in the Brazilian population used low‐resolution typing, generally restricted to the identification of a single allelic cluster, which limits more precise characterisation of associations. Thus, our findings offer a unique and detailed view of the role of HLA and non‐HLA variants in celiac disease susceptibility in the Brazilian context, confirming previous results regarding HLA‐DQ2.5 and HLA‐DQ2.2, as well as a possible role for the NFIA protein.

## Author Contributions

Conceptualisation, A.B.W.B.; methodology, F.V.da.S., V.B.‐B.H., P.I.dos.S., L.C.O., N.C., M.W., A.F., P.J.N., A.B.W.B.; validation and visualisation, F.V.da.S., V.B.‐B.H., P.I.dos.S., N.C., M.W., A.F., P.J.N., A.B.W.B.; formal analysis and investigation, F.V.da.S., V.B.‐B.H., E.D.A., A.B.W.B.; writing: original draft preparation, F.V.da.S., V.B.‐B.H., A.B.W.B.; writing: review and editing, F.V.da.S., V.B.‐B.H., E.D.A., P.I.dos.S., N.C., L.C.O., J.E.H., M.W., A.F., P.J.N., A.B.W.B.; supervision and project administration, A.B.W.B.; funding acquisition, J.E.H., M.W., A.F., P.J.N., A.B.W.B. All authors have read and agreed to the published version of the manuscript.

## Funding

This work was supported by Conselho Nacional de Desenvolvimento Científico e Tecnológico, (423317/2021‐0, 444227/2023‐7, JDT2022271000049, 313741/2021‐2, 311230/2025‐3). National Institutes of Health, (U01 AI090905). Fundação Araucária, (SUS2020131000106). Coordinação de Aperfeiçoamento de Pessoal de Nível Superior, PROAP‐01, Finance Code 001.

## Conflicts of Interest

The authors declare no conflicts of interest.

## Supporting information


**Table S1:** CeD predisposition SNPs selected for IPLEX.
**Table S2:** Multivariate analysis of HLA allele frequencies.
**Table S3:** Frequency of individuals carrying HLA‐DQ heterodimers at lower resolution.
**Table S4:** Distribution of HLA‐DQ heterodimer haplotypes at lower resolution.
**Table S5:** Analysis of SNPs associated with CeD.

## Data Availability

The data that support the findings of this study are available on request from the corresponding author. The data are not publicly available due to privacy or ethical restrictions.
