# Supplementary material for: Genetics of Celiac Disease in Southern Brazil: High‐Resolution HLA Haplotypes and Non‐HLA Polymorphisms
Source: HLA. 2026 Sep 26;108(3):e70972. doi: 10.1111/tan.70972 (PMC13615425; doi:10.1111/tan.70972)
Supplement: Supplementary file 1 [file TAN-108-e70972-s001.docx]

**Supplementary Tables**

**Supplementary table 1 - CeD predisposition SNPs selected for IPLEX.**

| **Genes** | **Description¹** | **SNP** | **Reference** |
| --- | --- | --- | --- |
| *PUS10* | Regulates genes involved in apoptosis, inflammation, immune response and oncogenic processes. | *rs13003464-G* | Dubois, *et al.* (2010)  Trynka *et al.* (2011) |
| *BLTP1* | This gene is located on the long arm of chromosome 4 in a region that is associated with susceptibility to CeD. | *rs13151961-G* | Dubois, *et al.* (2010)  Garner, *et al.* (2015) |
| *IL12A* | This gene encodes a subunit of a cytokine that acts on T and natural killer cells. | *rs1353248-T* | Trynka, *et al.* (2011)  Márquez, *et al.* (2018) |
| *IL18RAP, SLC9A4* | *IL18RAP:* The encoded protein is a pro-inflammatory cytokine involved in the induction of mediated immunity.  *SLC9A4:* Predicted to be involved in potassium ion transmembrane transport. | *rs990171-A* | Trynka, *et al.* (2011) |
| *RGS1* | The protein attenuates G protein signaling activity by binding to GTP-bound alpha G subunits and acting as a GTPase-activating protein. | *rs2816316-C* | Hunt, *et al.* (2008)  Dubois, *et al.* (2010) |
|  |  | *rs1359062-C* | Trynka, *et al.* (2011)  Márquez, *et al.* (2018) |
| *LINC03004* | It is an RNA gene, and is affiliated with the lncRNA class. | *rs17264332-G* | Trynka, *et al.* (2011)  Gutierrez-Achury, *et al.* (2019) |
| *AIMP1P2, TNFSF18* | The protein encoded by this gene is a cytokine belonging to the family of tumor necrosis factor (TNF) ligands. | *rs12142280-A* | Trynka, *et al.* (2011) |
| *TAGAP* | The encoded protein may function as a Rho GTPase activating protein. | *rs1738074-C* | Hunt, *et al.* (2008)  Dubois, *et al.* (2010) |
|  |  | *rs182429-A* | Trynka, *et al.* (2011) |
| *UBASH3A* | This family member can facilitate apoptosis induced by growth factor withdrawal in T cells. | *rs1893592-C* | Trynka, *et al.* (2011)  Gutierrez-Achury, *et al.* (2019)  Márquez, *et al.* (2018) |
| *ATXN2* | Mutations in this gene may be associated with susceptibility to type I diabetes, obesity and hypertension. | *rs653178-C* | Hunt, *et al.* (2008)  Dubois, *et al.* (2010) |
| *ZMIZ1* | The encoded protein regulates the activity of several transcription factors. | *rs1250552-G* | Dubois, *et al.* (2010)  Trynka, *et al. (*2011) |
| *YDJC,*  *UBE2L3* | *YDJC:* predicted to enable deacetylase activity and magnesium ion binding activity. Predicted to be involved in carbohydrate metabolic process.  *UBE2L3*: modification of proteins with ubiquitin targets abnormal or short-lived proteins for degradation. | *rs4821124-C* | Trynka, *et al.* (2011)  Gutierrez-Achury, *et al.* (2019) |
| *NFIA* | This gene encodes a member of the NF1 family of transcription factors. | *rs6691768-G* | Dubois, *et al.* (2010)  Márquez, *et al.* (2018) |
| *MIR4491,*  *POU2AF3* | *MIR4491*: non-coding RNAs that are involved in post-transcriptional regulation of gene expression  *POU2AF3:* involved in the positive regulation of transcription by RNA polymerase II. | *rs7104791-T* | Trynka, *et al.* (2011)  Márquez, *et al.* (2018) |

*¹SOURCE: NCBI - National Center for Biotechnology Information and GWAS Catalog. LEGEND: NFIA - nuclear factor I A; AIMP1P2 - aminoacyl tRNA synthetase complex interacting multifunctional protein 1 pseudogene 2, TNFSF18 - TNF superfamily member 18; RGS1 - regulator of G protein signaling 1; PUS10 - pseudouridine synthase 10; SLC9A4 - solute carrier family 9 member A4, IL18RAP - interleukin 18 receptor accessory protein; IL12A-AS1 - IL12A antisense RNA 1; BLTP1 - bridge-like lipid transfer protein family member 1; LINC03004 - long intergenic non-protein coding RNA 3004; TAGAP-AS1 - TAGAP antisense RNA 1, TAGAP - T cell activation RhoGTPase activating protein; ZMIZ1 - zinc finger MIZ-type containing 1; MIR4491 - microRNA 4491, POU2AF3 - POU class 2 homeobox associating factor 3; ATXN2 - ataxin 2; UBASH3A - ubiquitin associated and SH3 domain containing A; UBE2L3 - ubiquitin conjugating enzyme E2 L3, YDJC - YdjC chitooligosaccharide deacetylase homolog.*

**Supplementary table 2 - Multivariate analysis of *HLA* allele frequencies.**

|  | **Dominant model** | | | | **Additive model** | | | | **Recessive model** | | | |
| --- | --- | --- | --- | --- | --- | --- | --- | --- | --- | --- | --- | --- |
| ***HLA* ALLELE** | **OR** | **95% CI** | **p** | **pcorr** | **OR** | **95% CI** | **p** | **pcorr^1^** | **OR** | **95% CI** | **p** | **pcorr** |
| *A*01:01:01* | 2.53 | 1.54 - 4.22 | 2.9e-04 | **5.5e-04** | 2.10 | 1.35 - 3.32 | 0.001 | **0.002** | 1.22 | 0.28 - 5.39 | 0.78 | 0.87 |
| *B*08:01:01* | 4.08 | 2.40 - 7.16 | 4.3e-07 | **1.5e-06** | 3.44 | 2.11 - 5.80 | 1.6e-06 | **5.4e-06** | 3.44 | 0.77 - 24.0 | 0.14 | 0.49 |
| *C*07:01:01* | 2.52 | 1.58 - 4.06 | 1.2e-04 | **2.6e-04** | 2.23 | 1.47 - 3.44 | 2.2e-04 | **4.7e-04** | 2.35 | 0.64 - 11.1 | 0.22 | 0.49 |
| *DPB1*01:01:01* | 2.06 | 1.25 - 3.44 | 0.005 | **0.006** | 1.96 | 1.26 - 3.12 | 0.003 | **0.005** | 3.93 | 0.86 - 27.8 | 0.10 | 0.49 |
| *DPB1*17:01:01* | 4.27 | 1.66 - 13.2 | 0.005 | **0.006** | 4.27 | 1.66 - 13.2 | 0.005 | **0.006** | - | - | - | - |
| *DQA1*01:02:01* | 0.44 | 0.26 - 0.71 | 0.001 | **0.002** | 0.43 | 0.26 - 0.68 | 5.0e-04 | **9.5e-04** | - | - | - | - |
| *DQA1*02:01:01* | 2.88 | 1.80 - 4.68 | 1.3e-05 | **3.7e-05** | 2.49 | 1.60 - 3.92 | 6.1e-05 | **1.7e-04** | 0.91 | 0.12 - 5.83 | 0.92 | 0.92 |
| *DQA1*04:01:01* | 0.05 | 0.00 - 0.22 | 0.003 | **0.004** | 0.05 | 0.00 - 0.25 | 0.004 | **0.005** | - | - | - | - |
| *DQA1*05:01:01* | 4.94 | 3.08 - 8.06 | 6.6e-11 | **3.8e-10** | 3.64 | 2.39 - 5.68 | 4.7e-09 | **2.7e-08** | 1.94 | 0.65 - 6.47 | 0.25 | 0.49 |
| *DQB1*02:01:01* | 5.03 | 3.13 - 8.23 | 5.1e-11 | **3.8e-10** | 3.69 | 2.42 - 5.79 | 4.4e-09 | **2.7e-08** | 1.72 | 0.56 - 5.83 | 0.35 | 0.59 |
| *DQB1*02:02:01* | 3.42 | 2.14 - 5.56 | 4.2e-07 | **1.5e-06** | 3.06 | 1.96 - 4.86 | 1.4e-06 | **5.4e-06** | 1.79 | 0.29 - 14.0 | 0.54 | 0.76 |
| *DQB1*04:02:01* | 0.09 | 0.01 - 0.30 | 0.001 | **0.002** | 0.10 | 0.02 - 0.33 | 0.002 | **0.003** | - | - | - | - |
| *DQB1*05:01:01* | 0.44 | 0.23 - 0.80 | 0.009 | **0.010** | 0.44 | 0.23 - 0.78 | 0.006 | **0.007** | - | - | - | - |
| *DRB1*03:01:01* | 5.23 | 3.24 - 8.61 | 2.9e-11 | **3.8e-10** | 3.79 | 2.47 - 5.96 | 2.7e-09 | **2.7e-08** | 1.94 | 0.65 - 6.47 | 0.25 | 0.49 |
| *DRB1*07:01:01* | 2.72 | 1.70 - 4.40 | 3.5e-05 | **8.6e-05** | 2.35 | 1.51 - 3.70 | 1.7e-04 | **4.1e-04** | 0.77 | 0.10 - 4.79 | 0.77 | 0.87 |
| *DRB1*08:02:01* | 0.07 | 0.00 - 0.38 | 0.012 | **0.012** | 0.07 | 0.00 - 0.38 | 0.012 | **0.012** | - | - | - | - |
| *DRB1*13:01:01* | 0.40 | 0.20 - 0.78 | 0.010 | **0.010** | 0.40 | 0.20 - 0.74 | 0.006 | **0.007** | - | - | - | - |

*Legend: Logistic regression association tests were done with allele frequencies (additive model), frequency of homozygotes for the minor allele (recessive model), and summed frequencies of heterozygotes and homozygotes for the minor allele (dominant model). OR, odds ratio; CI, confidence interval; p, p-value; pcorr, corrected p-values using “False discovery rate” method. In bold, significant associations (p < 0.05).*

**Supplementary table 3 - Frequency of individuals carrying HLA-DQ heterodimers at lower resolution.**

| **HETERODIMER** | **DQA1** | **DQB1** | **CeD**  **N = 174 (%)** | **CTR**  **N = 196 (%)** | **OR** | **95% CI** | **pcorr** |
| --- | --- | --- | --- | --- | --- | --- | --- |
| **DQ2.5** | ***DQA1*05*** | ***DQB1*02*** | **115 (66.1)** | **46 (23.5)** | **6.36** | **4.06, 10.1** | **<0.001** |
| **DQ2.2** | ***DQA1*02*** | ***DQB1*02*** | **71 (40.8)** | **33 (16.8)** | **3.40** | **2.12, 5.56** | **<0.001** |
| DQ2.3 | *DQA1*03* | *DQB1*02* | 17 (9.8) | 11 (5.6) | 1.82 | 0.84, 4.12 | 0.23 |
| DQ9 | *DQA1*03* | *DQB1*03:03* | 3 (1.7) | 8 (4.1) | 0.41 | 0.09, 1.45 | 0.31 |
| DQ8 | *DQA1*03* | *DQB1*03:02* | 35 (20.1) | 36 (18.4) | 1.12 | 0.67, 1.88 | 0.75 |
| DQ7.5 | *DQA1*05:05* | *DQB1*03:01* | 43 (24.7) | 49 (25) | 0.98 | 0.61, 1.58 | 0.95 |
| **Absents** | | | **7 (4.0)** | **59 (30.1)** | **0.10** | **0.04, 0.21** | **<0.001** |

*Legend: CTR: controls; CeD: case; DQA1: alpha chain; DQB1: beta chain; OR, odds ratio; CI, confidence interval; pcorr, corrected p-values using “False discovery rate” method. In bold, significant associations (p < 0.05); Absents: Individuals without the above alleles.*

**Supplementary table 4 - Distribution of *HLA-DQ* heterodimer haplotypes at lower resolution.**

| **HETERODIMER** | **CeD**  **N = 174 (%)** | **CTR**  **N = 196 (%)** | **OR** | **95% CI** | **pcorr** |
| --- | --- | --- | --- | --- | --- |
| **DQ2.5** | **48 (27.6)** | **28 (14.3)** | **2.29** | **1.37 - 3.88** | **0.007** |
| DQ2.2 | 10 (5.7) | 18 (9.2) | 0.60 | 0.26 - 1.32 | 0.41 |
| DQ2.3 | 1 (0.6) | 2 (1.0) | 0.56 | 0.03 - 5.90 | 0.75 |
| **DQ7.5** | **7 (4.0)** | **33 (16.8)** | **0.21** | **0.08 - 0.46** | **0.001** |
| DQ8 | 17 (9.8) | 22 (11.2) | 0.86 | 0.43 - 1.67 | 0.75 |
| DQ9 | 2 (1.1) | 3 (1.5) | 0.75 | 0.10 - 4.56 | 0.80 |
| ***DQ2.2~2.5**** | **30 (17.2)** | **3 (1.5)** | **13.4** | **4.66 - 56.7** | **<0.001** |
| *DQ2.2~2.3~8** | 7 (4.0) | 5 (2.6) | 1.60 | 0.50 - 5.50 | 0.58 |
| ***DQ2.2~2.5~7.5**** | **22 (12.6)** | **7 (2.6)** | **3.91** | **1.70 - 10.1** | **0.007** |
| *DQ2.3~2.5~8** | 5 (2.9) | 3 (1.5) | 1.90 | 0.46 - 9.39 | 0.58 |
| *DQ2.5~7.5** | 9 (5.2) | 4 (2.0) | 2.62 | 0.84 - 9.81 | 0.25 |
| Others* | 9 (5.2) | 9 (4.6) | 1.13 | 0.43 - 2.97 | 0.80 |
| ***Absents*** | **7 (4.0)** | **59 (30.1)** | **0.10** | **0.04 - 0.21** | **<0.001** |

*Legend: CTR: controls; CeD: case; DQA1: alpha chain; DQB1: beta chain; OR, odds ratio; CI, confidence interval; pcorr, corrected p-values using “False discovery rate” method. In bold, significant associations (p < 0.05); Absents: Individuals without the above alleles; *in trans: The individual exhibits more than one type of heterodimer.*

**Supplementary table 5 - Analysis of SNPs associated with CeD.**

| **GENE** | **SNP** | **MAF (%)** | | **MODEL** | **CTR** | **CeD** | **OR** | **95% CI** | ***p*** | ***pcorr*** |
| --- | --- | --- | --- | --- | --- | --- | --- | --- | --- | --- |
|  |  | **CTR** | **CeD** |  |  |  |  |  |  |  |
| *NFIA* | *rs6691768* | 42.3 | 34.2 | add | 42.3 | 34.2 | 0.68 | .046 - 1.00 | 0.053 | 0.066 |
| 1p31.3 | g < A |  |  | rec | 15.4 | 12.9 | 0.80 | 0.37 - 1.69 | 0.6 | 0.6 |
|  | Intron variant |  |  | **dom** | **69.2** | **55.5** | **0.53** | **0.31 - 0.92** | **0.024** | **0.039** |
| *AIMP1P2, TNFSF18* | *rs12142280* | 17.2 | 17.7 | add | 17.2 | 17.7 | 1.04 | 0.65 - 1.65 | 0.9 | 0.9 |
| 1q24.3 | a < T |  |  | rec | 3.8 | 4.5 | 1.89 | 0.52 - 7.06 | 0.3 | 0.4 |
|  | Intron variant |  |  | dom | 30.6 | 31.0 | 0.93 | 0.53 - 1.63 | 0.8 | 0.8 |
| *RGS1* | *rs2816316* | 17.2 | 20.2 | add | 17.2 | 20.0 | 1.13 | 0.71 - 1.80 | 0.6 | 0.6 |
| 1q31.2 | c < A |  |  | rec | 1.9 | 5.8 | 2.71 | 0.67 - 13.8 | 0.2 | 0.2 |
|  | Intron variant |  |  | dom | 32.5 | 34.2 | 1.01 | 0.59 - 1.75 | >0.9 | >0.9 |
| *PUS10, REL* | *rs13003464* | 43.0 | 49.4 | add | 43.0 | 49.4 | 1.40 | 0.98 - 2.03 | 0.069 | 0.086 |
| *2p16.1* | g < A |  |  | rec | 17.7 | 26.5 | 1.89 | 1.01 - 3.58 | 0.049 | 0.061 |
|  | Intron variant |  |  | dom | 68.4 | 72.3 | 1.36 | 0.77 - 2.40 | 0.3 | 0.4 |
| *SLC9A4, IL18RAP* | *rs990171* | 29.9 | 26.1 | add | 29.9 | 26.1 | 0.90 | 0.59 - 1.37 | 0.6 | 0.6 |
| 2q12.1 | a < C |  |  | rec | 10.2 | 5.8 | 0.62 | 0.21 - 1.73 | 0.4 | 0.5 |
|  | Intergenic variant |  |  | dom | 49.7 | 46.5 | 0.96 | 0.57 - 1.62 | 0.9 | 0.9 |
| *IL12A-AS1* | *rs1353248* | 22.6 | 25.8 | add | 22.6 | 25.8 | 1.23 | 0.82 - 1.85 | 0.3 | 0.4 |
| 3q25.33 | t < C |  |  | rec | 5.7 | 9.0 | 1.50 | 0.57 - 4.16 | 0.4 | 0.5 |
|  | Intergenic variant |  |  | dom | 39.5 | 42.6 | 1.25 | 0.74 - 2.10 | 0.4 | 0.5 |
| *BLTP1* | *rs13151961* | 11.9 | 10.0 | add | 11.9 | 10.0 | 0.62 | 0.33 - 1.14 | 0.12 | 0.2 |
| 4q27 | g < A |  |  | rec | 64.1 | 64.5 | 0.47 | 0.01 - 22.1 | 0.7 | 0.7 |
|  | Intron variant |  |  | dom | 23.1 | 19.4 | 0.61 | 0.32 - 1.14 | 0.13 | 0.2 |
| *LINC03004* | *rs17264332* | 19.6 | 22.6 | add | 19.6 | 22.6 | 1.32 | 0.85 - 2.04 | 0.2 | 0.3 |
| 6q23.3 | g < A |  |  | rec | 4.5 | 6.5 | 1.52 | 0.50 - 4.81 | 0.5 | 0.5 |
|  | Intron variant |  |  | dom | 34.6 | 38.7 | 1.38 | 0.81 - 2.38 | 0.2 | 0.3 |
|  | *rs1738074* | 50.6 | 49.8 | add | 50.6 | 49.7 | 0.98 | 0.68 - 1.40 | 0.9 | 0.9 |
|  | c < T |  |  | rec | 26.8 | 25.2 | 0.96 | 0.53 - 1.72 | 0.9 | 0.9 |
| *TAGAP-AS1, TAGAP* | 5 prime UTR variant |  |  | dom | 74.5 | 74.2 | 0.98 | 0.54 - 1.77 | >0.9 | >0.9 |
| 6q25.3 | *rs182429* | 49.7 | 50.1 | add | 49.7 | 51.0 | 1.03 | 0.72 - 1.48 | 0.9 | 0.9 |
|  | a < G |  |  | rec | 25.5 | 26.5 | 1.05 | 0.58 - 1.90 | 0.9 | 0.9 |
|  | Intron variant |  |  | dom | 73.9 | 75.5 | 1.03 | 0.57 - 1.85 | >0.9 | >0.9 |
| *ZMIZ1* | *rs1250552* | 51.6 | 44.8 | add | 51.6 | 44.8 | 0.68 | 0.47 - 0.99 | 0.047 | 0.059 |
| 10q22.3 | g < A |  |  | rec | 24.8 | 20.6 | 0.67 | 0.36 - 1.25 | 0.2 | 0.3 |
|  | Intron variant |  |  | dom | 78.3 | 69.0 | 0.55 | 0.30 - 0.99 | 0.050 | 0.062 |
| *MIR4491, POU2AF3* | *rs7104791* | 22.3 | 19.7 | add | 22.3 | 19.7 | 0.75 | 0.47 - 1.20 | 0.2 | 0.3 |
| 11q23.1 | t < C |  |  | rec | 4.5 | 2.6 | 0.49 | 0.10 - 1.98 | 0.3 | 0.4 |
|  | Intergenic variant |  |  | dom | 40.1 | 36.8 | 0.76 | 0.44 - 1.30 | 0.3 | 0.4 |
| *ATXN2* | *rs653178* | 38.9 | 49.7 | **add** | **38.9** | **49.7** | **1.49** | **1.03 - 2.16** | **0.033** | **0.042** |
| 12q24.12 | c < T |  |  | rec | 15.3 | 25.8 | 1.69 | 0.88 - 3.31 | 0.12 | 0.14 |
|  | Intron variant |  |  | dom | 62.4 | 73.5 | 1.71 | 0.99 - 2.99 | 0.057 | 0.072 |
| *UBASH3A* | *rs1893592* | 20.7 | 19.5 | add | 20.7 | 19.5 | 1.03 | 0.66 - 1.60 | 0.9 | 0.9 |
| 21q22.3 | c < A |  |  | rec | 7.0 | 2.6 | 0.38 | 0.10 - 1.29 | 0.14 | 0.2 |
|  | Splice donor region variant |  |  | dom | 34.4 | 36.4 | 1.27 | 0.74 - 2.18 | 0.4 | 0.5 |
| *UBE2L3, YDJC* | *rs4821124* | 19.7 | 22.9 | add | 19.7 | 22.9 | 1.42 | 0.91 - 2.24 | 0.12 | 0.2 |
| 22q11.21 | c < T |  |  | rec | 5.7 | 3.9 | 0.82 | 0.23 - 2.76 | 0.7 | 0.7 |
|  | Regulatory region variant |  |  | dom | 33.8 | 41.9 | 1.73 | 1.01 - 2.97 | 0.047 | 0.058 |

*Logistic regression association tests were done with allele frequencies (“add”), frequency of homozygotes for the minor allele (“rec”- recessive model), and summed frequencies of heterozygotes and homozygotes for the minor allele (“dom”—dominant model). Sample size slightly differed for controls (N=156-158 individuals) and patients (N=154-155 individuals) because of genotyping quality. The minor allele in our sample is given in lowercase; being the reference for the associations. In bold, significant associations (pcorr < 0.05). SNP, single nucleotide polymorphism; MAF, minor allele frequency; CTR controls; CeD, patients; Model, association tests; OR, odds ratio; CI, confidence interval; pcorr: corrected p-values using “False discovery rate” method;*
